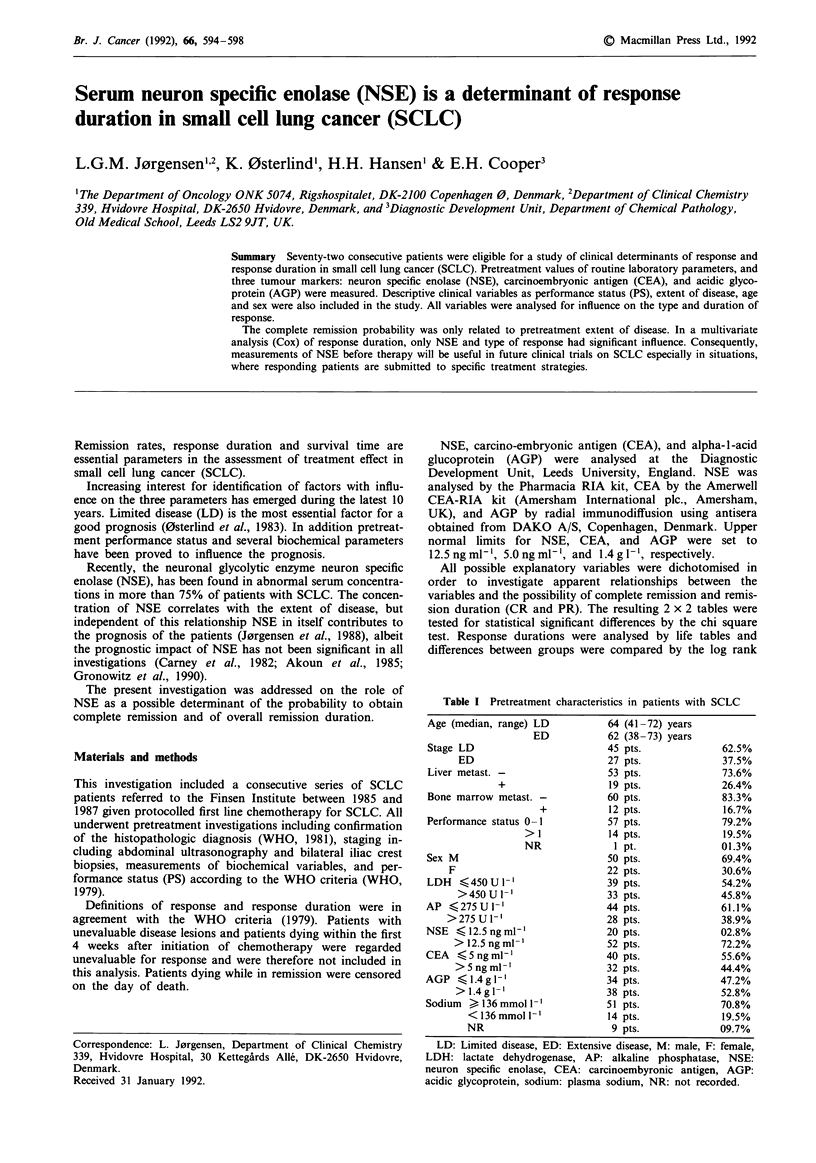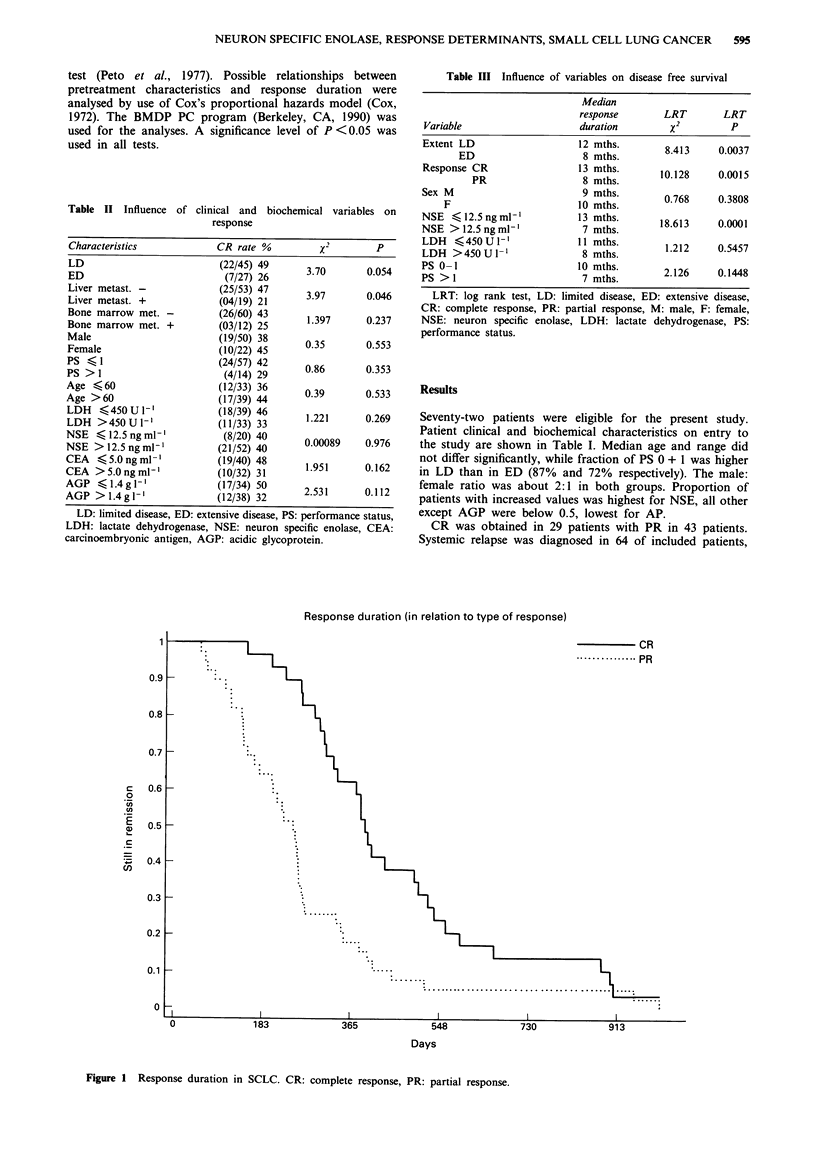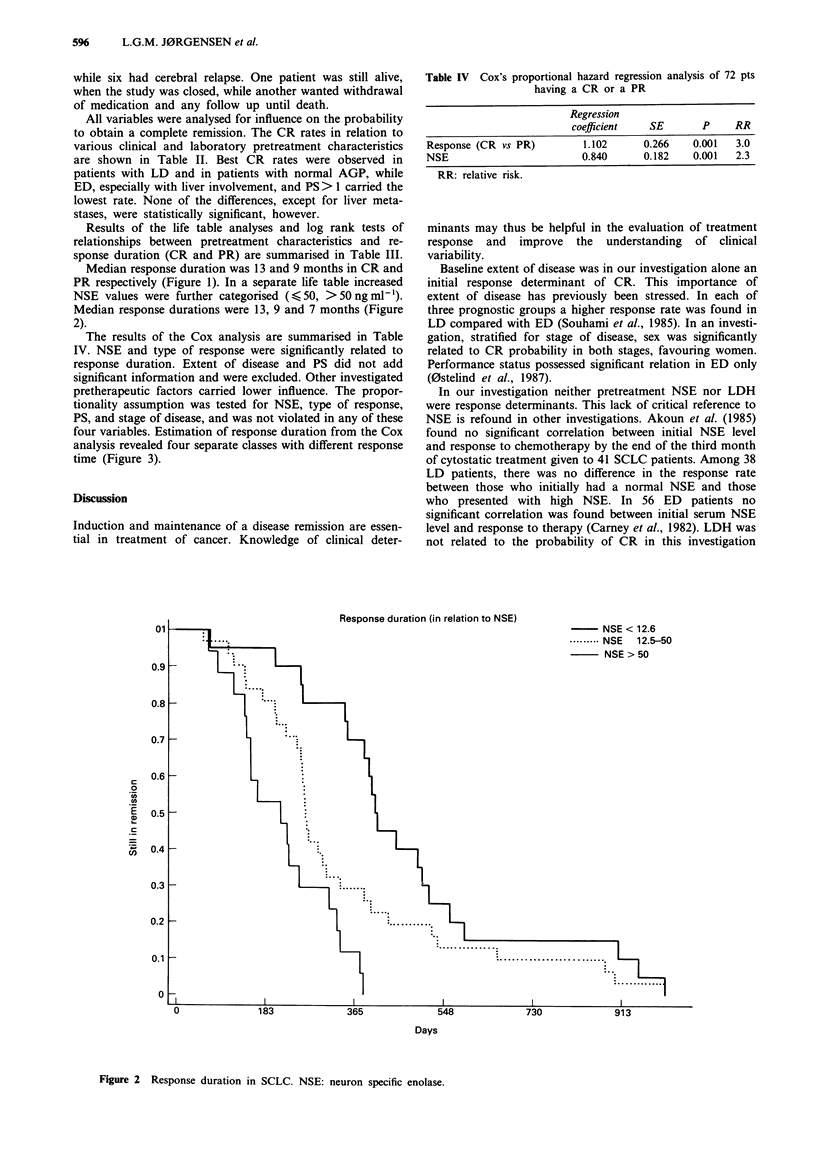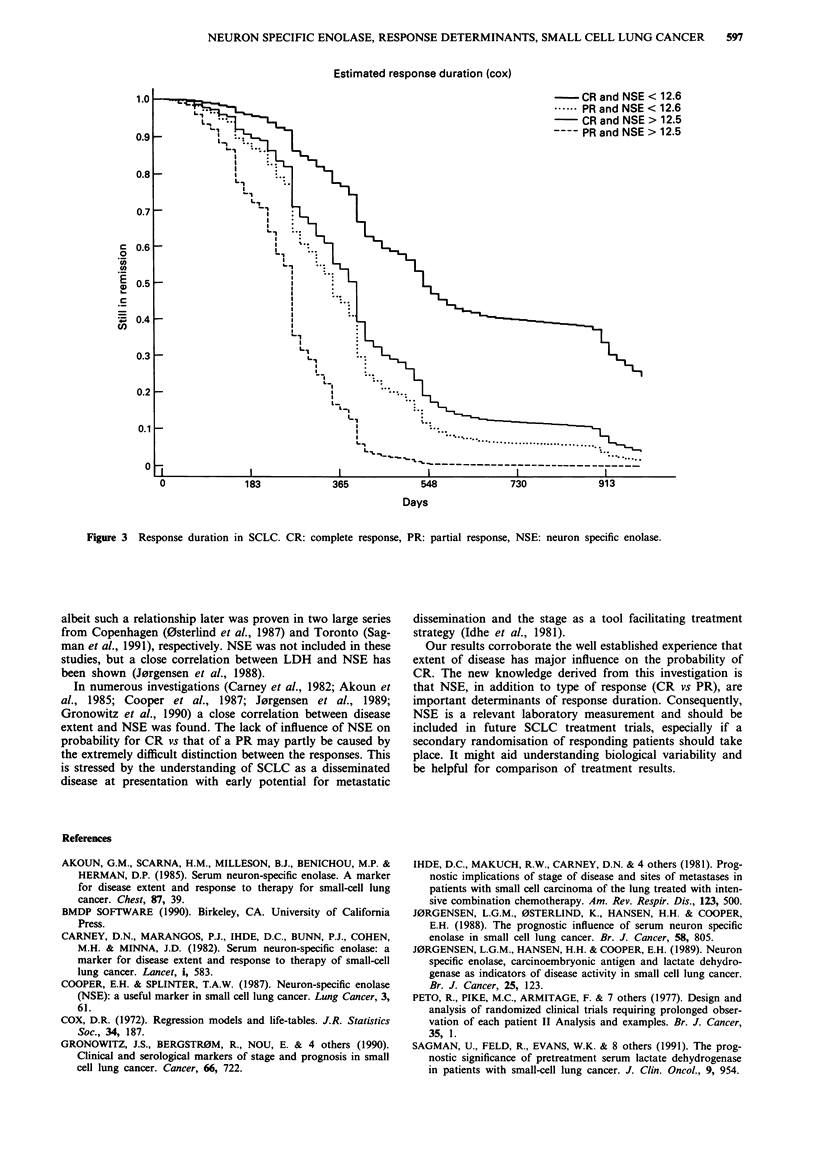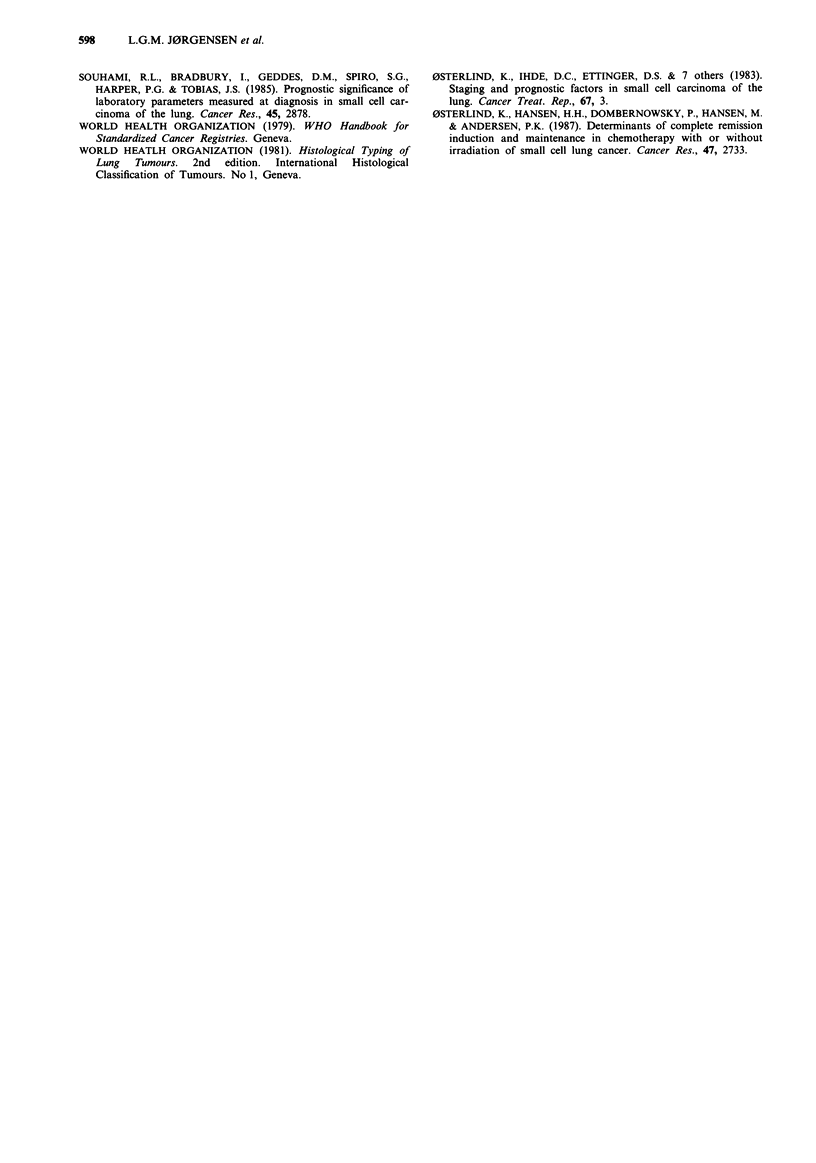# Serum neuron specific enolase (NSE) is a determinant of response duration in small cell lung cancer (SCLC).

**DOI:** 10.1038/bjc.1992.320

**Published:** 1992-09

**Authors:** L. G. Jørgensen, K. Osterlind, H. H. Hansen, E. H. Cooper

**Affiliations:** Department of Oncology Rigshospitalet, Copenhagen, Denmark.

## Abstract

Seventy-two consecutive patients were eligible for a study of clinical determinants of response and response duration in small cell lung cancer (SCLC). Pretreatment values of routine laboratory parameters, and three tumour markers: neuron specific enolase (NSE), carcinoembryonic antigen (CEA), and acidic glycoprotein (AGP) were measured. Descriptive clinical variables as performance status (PS), extent of disease, age and sex were also included in the study. All variables were analysed for influence on the type and duration of response. The complete remission probability was only related to pretreatment extent of disease. In a multivariate analysis (Cox) of response duration, only NSE and type of response had significant influence. Consequently, measurements of NSE before therapy will be useful in future clinical trials on SCLC especially in situations, where responding patients are submitted to specific treatment strategies.


Br. J. Cancer (1992), 66, 594-598 © Macmillan Press Ltd., 1992

### Serum neuron specific enolase (NSE) is a determinant of response duration in small cell lung cancer (SCLC)

L.G.M. Jørgensen[1,2], K. Østerlind[1], H.H. Hansen[1] & E.H. Cooper[3]

[1]*The Department of Oncology ONK 5074, Rigshospitalet, DK-2100 Copenhagen Ø, Denmark,* [2]*Department of Clinical Chemistry 339, Hvidovre Hospital, DK-2650 Hvidovre, Denmark, and* [3]*Diagnostic Development Unit, Department of Chemical Pathology, Old Medical School, Leeds LS2 9JT, UK.*

**Summary** Seventy-two consecutive patients were eligible for a study of clinical determinants of response and response duration in small cell lung cancer (SCLC). Pretreatment values of routine laboratory parameters, and three tumour markers: neuron specific enolase (NSE), carcinoembryonic antigen (CEA), and acidic glycoprotein (AGP) were measured. Descriptive clinical variables as performance status (PS), extent of disease, age and sex were also included in the study. All variables were analysed for influence on the type and duration of response.

The complete remission probability was only related to pretreatment extent of disease. In a multivariate analysis (Cox) of response duration, only NSE and type of response had significant influence. Consequently, measurements of NSE before therapy will be useful in future clinical trials on SCLC especially in situations, where responding patients are submitted to specific treatment strategies.

Remission rates, response duration and survival time are essential parameters in the assessment of treatment effect in small cell lung cancer (SCLC).

Increasing interest for identification of factors with influence on the three parameters has emerged during the latest 10 years. Limited disease (LD) is the most essential factor for a good prognosis (Østerlind *et al.*, 1983). In addition pretreatment performance status and several biochemical parameters have been proved to influence the prognosis.

Recently, the neuronal glycolytic enzyme neuron specific enolase (NSE), has been found in abnormal serum concentrations in more than 75% of patients with SCLC. The concentration of NSE correlates with the extent of disease, but independent of this relationship NSE in itself contributes to the prognosis of the patients (Jørgensen *et al.*, 1988), albeit the prognostic impact of NSE has not been significant in all investigations (Carney *et al.*, 1982; Akoun *et al.*, 1985; Gronowitz *et al.*, 1990).

The present investigation was addressed on the role of NSE as a possible determinant of the probability to obtain complete remission and of overall remission duration.

### Materials and methods

This investigation included a consecutive series of SCLC patients referred to the Finsen Institute between 1985 and 1987 given protocolled first line chemotherapy for SCLC. All underwent pretreatment investigations including confirmation of the histopathologic diagnosis (WHO, 1981), staging including abdominal ultrasonography and bilateral iliac crest biopsies, measurements of biochemical variables, and performance status (PS) according to the WHO criteria (WHO, 1979).

Definitions of response and response duration were in agreement with the WHO criteria (1979). Patients with unevaluable disease lesions and patients dying within the first 4 weeks after initiation of chemotherapy were regarded unevaluable for response and were therefore not included in this analysis. Patients dying while in remission were censored on the day of death.

Correspondence: L. Jørgensen, Department of Clinical Chemistry 339, Hvidovre Hospital, 30 Kettegårds Allé, DK-2650 Hvidovre, Denmark.
Received 31 January 1992.

NSE, carcino-embryonic antigen (CEA), and alpha-1-acid glucoprotein (AGP) were analysed at the Diagnostic Development Unit, Leeds University, England. NSE was analysed by the Pharmacia RIA kit, CEA by the Amerwell CEA-RIA kit (Amersham International plc., Amersham, UK), and AGP by radial immunodiffusion using antisera obtained from DAKO A/S, Copenhagen, Denmark. Upper normal limits for NSE, CEA, and AGP were set to 12.5 ng $ml^{-1}$, 5.0 ng $ml^{-1}$, and 1.4 g $l^{-1}$, respectively.

All possible explanatory variables were dichotomised in order to investigate apparent relationships between the variables and the possibility of complete remission and remission duration (CR and PR). The resulting 2 × 2 tables were tested for statistical significant differences by the chi square test. Response durations were analysed by life tables and differences between groups were compared by the log rank

**Table I** Pretreatment characteristics in patients with SCLC

| | | |
|---|---|---|
| Age (median, range) LD | 64 (41–72) years | |
| ED | 62 (38–73) years | |
| Stage LD | 45 pts. | 62.5% |
| ED | 27 pts. | 37.5% |
| Liver metast. – | 53 pts. | 73.6% |
| + | 19 pts. | 26.4% |
| Bone marrow metast. – | 60 pts. | 83.3% |
| + | 12 pts. | 16.7% |
| Performance status 0–1 | 57 pts. | 79.2% |
| >1 | 14 pts. | 19.5% |
| NR | 1 pt. | 01.3% |
| Sex M | 50 pts. | 69.4% |
| F | 22 pts. | 30.6% |
| LDH ⩽450 U $l^{-1}$ | 39 pts. | 54.2% |
| >450 U $l^{-1}$ | 33 pts. | 45.8% |
| AP ⩽275 U $l^{-1}$ | 44 pts. | 61.1% |
| >275 U $l^{-1}$ | 28 pts. | 38.9% |
| NSE ⩽12.5 ng $ml^{-1}$ | 20 pts. | 02.8% |
| >12.5 ng $ml^{-1}$ | 52 pts. | 72.2% |
| CEA ⩽5 ng $ml^{-1}$ | 40 pts. | 55.6% |
| >5 ng $ml^{-1}$ | 32 pts. | 44.4% |
| AGP ⩽1.4 g $l^{-1}$ | 34 pts. | 47.2% |
| >1.4 g $l^{-1}$ | 38 pts. | 52.8% |
| Sodium ⩾136 mmol $l^{-1}$ | 51 pts. | 70.8% |
| <136 mmol $l^{-1}$ | 14 pts. | 19.5% |
| NR | 9 pts. | 09.7% |

LD: Limited disease, ED: Extensive disease, M: male, F: female, LDH: lactate dehydrogenase, AP: alkaline phosphatase, NSE: neuron specific enolase, CEA: carcinoembryonic antigen, AGP: acidic glycoprotein, sodium: plasma sodium, NR: not recorded.

test (Peto *et al.*, 1977). Possible relationships between pretreatment characteristics and response duration were analysed by use of Cox's proportional hazards model (Cox, 1972). The BMDP PC program (Berkeley, CA, 1990) was used for the analyses. A significance level of $P < 0.05$ was used in all tests.

**Table II** Influence of clinical and biochemical variables on response

| *Characteristics* | *CR rate %* | $\chi^2$ | *P* |
|---|---|---|---|
| LD | (22/45) 49 | 3.70 | 0.054 |
| ED | (7/27) 26 | | |
| Liver metast. – | (25/53) 47 | 3.97 | 0.046 |
| Liver metast. + | (04/19) 21 | | |
| Bone marrow met. – | (26/60) 43 | 1.397 | 0.237 |
| Bone marrow met. + | (03/12) 25 | | |
| Male | (19/50) 38 | 0.35 | 0.553 |
| Female | (10/22) 45 | | |
| PS $\leqslant 1$ | (24/57) 42 | 0.86 | 0.353 |
| PS $>1$ | (4/14) 29 | | |
| Age $\leqslant 60$ | (12/33) 36 | 0.39 | 0.533 |
| Age $>60$ | (17/39) 44 | | |
| LDH $\leqslant 450\ U\ l^{-1}$ | (18/39) 46 | 1.221 | 0.269 |
| LDH $>450\ U\ l^{-1}$ | (11/33) 33 | | |
| NSE $\leqslant 12.5\ ng\ ml^{-1}$ | (8/20) 40 | 0.00089 | 0.976 |
| NSE $>12.5\ ng\ ml^{-1}$ | (21/52) 40 | | |
| CEA $\leqslant 5.0\ ng\ ml^{-1}$ | (19/40) 48 | 1.951 | 0.162 |
| CEA $>5.0\ ng\ ml^{-1}$ | (10/32) 31 | | |
| AGP $\leqslant 1.4\ g\ l^{-1}$ | (17/34) 50 | 2.531 | 0.112 |
| AGP $>1.4\ g\ l^{-1}$ | (12/38) 32 | | |

LD: limited disease, ED: extensive disease, PS: performance status, LDH: lactate dehydrogenase, NSE: neuron specific enolase, CEA: carcinoembryonic antigen, AGP: acidic glycoprotein.

**Table III** Influence of variables on disease free survival

| *Variable* | *Median response duration* | *LRT* $\chi^2$ | *LRT P* |
|---|---|---|---|
| Extent LD | 12 mths. | 8.413 | 0.0037 |
| ED | 8 mths. | | |
| Response CR | 13 mths. | 10.128 | 0.0015 |
| PR | 8 mths. | | |
| Sex M | 9 mths. | 0.768 | 0.3808 |
| F | 10 mths. | | |
| NSE $\leqslant 12.5\ ng\ ml^{-1}$ | 13 mths. | 18.613 | 0.0001 |
| NSE $>12.5\ ng\ ml^{-1}$ | 7 mths. | | |
| LDH $\leqslant 450\ U\ l^{-1}$ | 11 mths. | 1.212 | 0.5457 |
| LDH $>450\ U\ l^{-1}$ | 8 mths. | | |
| PS 0–1 | 10 mths. | 2.126 | 0.1448 |
| PS $>1$ | 7 mths. | | |

LRT: log rank test, LD: limited disease, ED: extensive disease, CR: complete response, PR: partial response, M: male, F: female, NSE: neuron specific enolase, LDH: lactate dehydrogenase, PS: performance status.

### Results

Seventy-two patients were eligible for the present study. Patient clinical and biochemical characteristics on entry to the study are shown in Table I. Median age and range did not differ significantly, while fraction of PS 0 + 1 was higher in LD than in ED (87% and 72% respectively). The male: female ratio was about 2:1 in both groups. Proportion of patients with increased values was highest for NSE, all other except AGP were below 0.5, lowest for AP.

CR was obtained in 29 patients with PR in 43 patients. Systemic relapse was diagnosed in 64 of included patients,

**Figure 1** Response duration in SCLC. CR: complete response, PR: partial response.

while six had cerebral relapse. One patient was still alive, when the study was closed, while another wanted withdrawal of medication and any follow up until death.

All variables were analysed for influence on the probability to obtain a complete remission. The CR rates in relation to various clinical and laboratory pretreatment characteristics are shown in Table II. Best CR rates were observed in patients with LD and in patients with normal AGP, while ED, especially with liver involvement, and PS > 1 carried the lowest rate. None of the differences, except for liver metastases, were statistically significant, however.

Results of the life table analyses and log rank tests of relationships between pretreatment characteristics and response duration (CR and PR) are summarised in Table III.

Median response duration was 13 and 9 months in CR and PR respectively (Figure 1). In a separate life table increased NSE values were further categorised ($\leqslant 50$, $> 50 \text{ ng ml}^{-1}$). Median response durations were 13, 9 and 7 months (Figure 2).

The results of the Cox analysis are summarised in Table IV. NSE and type of response were significantly related to response duration. Extent of disease and PS did not add significant information and were excluded. Other investigated pretherapeutic factors carried lower influence. The proportionality assumption was tested for NSE, type of response, PS, and stage of disease, and was not violated in any of these four variables. Estimation of response duration from the Cox analysis revealed four separate classes with different response time (Figure 3).

### Discussion

Induction and maintenance of a disease remission are essential in treatment of cancer. Knowledge of clinical determinants may thus be helpful in the evaluation of treatment response and improve the understanding of clinical variability.

**Table IV** Cox's proportional hazard regression analysis of 72 pts having a CR or a PR

| | *Regression coefficient* | *SE* | *P* | *RR* |
|---|---|---|---|---|
| Response (CR *vs* PR) | 1.102 | 0.266 | 0.001 | 3.0 |
| NSE | 0.840 | 0.182 | 0.001 | 2.3 |

RR: relative risk.

Baseline extent of disease was in our investigation alone an initial response determinant of CR. This importance of extent of disease has previously been stressed. In each of three prognostic groups a higher response rate was found in LD compared with ED (Souhami *et al.*, 1985). In an investigation, stratified for stage of disease, sex was significantly related to CR probability in both stages, favouring women. Performance status possessed significant relation in ED only (Østelind *et al.*, 1987).

In our investigation neither pretreatment NSE nor LDH were response determinants. This lack of critical reference to NSE is refound in other investigations. Akoun *et al.* (1985) found no significant correlation between initial NSE level and response to chemotherapy by the end of the third month of cytostatic treatment given to 41 SCLC patients. Among 38 LD patients, there was no difference in the response rate between those who initially had a normal NSE and those who presented with high NSE. In 56 ED patients no significant correlation was found between initial serum NSE level and response to therapy (Carney *et al.*, 1982). LDH was not related to the probability of CR in this investigation

**Figure 2** Response duration in SCLC. NSE: neuron specific enolase.

**Figure 3** Response duration in SCLC. CR: complete response, PR: partial response, NSE: neuron specific enolase.

albeit such a relationship later was proven in two large series from Copenhagen (Østerlind *et al.*, 1987) and Toronto (Sagman *et al.*, 1991), respectively. NSE was not included in these studies, but a close correlation between LDH and NSE has been shown (Jørgensen *et al.*, 1988).

In numerous investigations (Carney *et al.*, 1982; Akoun *et al.*, 1985; Cooper *et al.*, 1987; Jørgensen *et al.*, 1989; Gronowitz *et al.*, 1990) a close correlation between disease extent and NSE was found. The lack of influence of NSE on probability for CR *vs* that of a PR may partly be caused by the extremely difficult distinction between the responses. This is stressed by the understanding of SCLC as a disseminated disease at presentation with early potential for metastatic dissemination and the stage as a tool facilitating treatment strategy (Idhe *et al.*, 1981).

Our results corroborate the well established experience that extent of disease has major influence on the probability of CR. The new knowledge derived from this investigation is that NSE, in addition to type of response (CR *vs* PR), are important determinants of response duration. Consequently, NSE is a relevant laboratory measurement and should be included in future SCLC treatment trials, especially if a secondary randomisation of responding patients should take place. It might aid understanding biological variability and be helpful for comparison of treatment results.

### References

AKOUN, G.M., SCARNA, H.M., MILLESON, B.J., BENICHOU, M.P. & HERMAN, D.P. (1985). Serum neuron-specific enolase. A marker for disease extent and response to therapy for small-cell lung cancer. *Chest*, **87**, 39.

BMDP SOFTWARE (1990). Birkeley, CA. University of California Press.

CARNEY, D.N., MARANGOS, P.J., IHDE, D.C., BUNN, P.J., COHEN, M.H. & MINNA, J.D. (1982). Serum neuron-specific enolase: a marker for disease extent and response to therapy of small-cell lung cancer. *Lancet*, **i**, 583.

COOPER, E.H. & SPLINTER, T.A.W. (1987). Neuron-specific enolase (NSE): a useful marker in small cell lung cancer. *Lung Cancer*, **3**, 61.

COX, D.R. (1972). Regression models and life-tables. *J.R. Statistics Soc.*, **34**, 187.

GRONOWITZ, J.S., BERGSTRØM, R., NOU, E. & 4 others (1990). Clinical and serological markers of stage and prognosis in small cell lung cancer. *Cancer*, **66**, 722.

IHDE, D.C., MAKUCH, R.W., CARNEY, D.N. & 4 others (1981). Prognostic implications of stage of disease and sites of metastases in patients with small cell carcinoma of the lung treated with intensive combination chemotherapy. *Am. Rev. Respir. Dis.*, **123**, 500.

JØRGENSEN, L.G.M., ØSTERLIND, K., HANSEN, H.H. & COOPER, E.H. (1988). The prognostic influence of serum neuron specific enolase in small cell lung cancer. *Br. J. Cancer*, **58**, 805.

JØRGENSEN, L.G.M., HANSEN, H.H. & COOPER, E.H. (1989). Neuron specific enolase, carcinoembryonic antigen and lactate dehydrogenase as indicators of disease activity in small cell lung cancer. *Br. J. Cancer*, **25**, 123.

PETO, R., PIKE, M.C., ARMITAGE, F. & 7 others (1977). Design and analysis of randomized clinical trials requiring prolonged observation of each patient II Analysis and examples. *Br. J. Cancer*, **35**, 1.

SAGMAN, U., FELD, R., EVANS, W.K. & 8 others (1991). The prognostic significance of pretreatment serum lactate dehydrogenase in patients with small-cell lung cancer. *J. Clin. Oncol.*, **9**, 954.

SOUHAMI, R.L., BRADBURY, I., GEDDES, D.M., SPIRO, S.G., HARPER, P.G. & TOBIAS, J.S. (1985). Prognostic significance of laboratory parameters measured at diagnosis in small cell carcinoma of the lung. *Cancer Res.*, **45**, 2878.

WORLD HEALTH ORGANIZATION (1979). *WHO Handbook for Standardized Cancer Registries*. Geneva.

WORLD HEATLH ORGANIZATION (1981). *Histological Typing of Lung Tumours*. 2nd edition. International Histological Classification of Tumours. No 1, Geneva.

ØSTERLIND, K., IHDE, D.C., ETTINGER, D.S. & 7 others (1983). Staging and prognostic factors in small cell carcinoma of the lung. *Cancer Treat. Rep.*, **67**, 3.

ØSTERLIND, K., HANSEN, H.H., DOMBERNOWSKY, P., HANSEN, M. & ANDERSEN, P.K. (1987). Determinants of complete remission induction and maintenance in chemotherapy with or without irradiation of small cell lung cancer. *Cancer Res.*, **47**, 2733.